# Spontaneous rupture and hematoma of the sartorius muscle secondary to rivaroxaban therapy

**DOI:** 10.1093/jscr/rjaa090

**Published:** 2020-04-24

**Authors:** Javier Ardebol, Mario Cahueque, Carlos Sanchez

**Affiliations:** 1 Medical Research, Universidad Francisco Marroquín, Guatemala, Guatemala; 2 Orthopedic Surgery, Hospital Centro Médico, Guatemala, Guatemala; 3 Cardiología Intervencionista, Hospital Centro Médico, Guatemala, Guatemala

**Keywords:** rivaroxaban, spontaneous hematoma, rupture, sartorius muscle

## Abstract

Spontaneous muscular hematomas are quite rare as they occur mush less frequently than intracranial hematomas and gastrointestinal bleeding in patients under oral anticoagulant therapy. Coumarins, such as warfarin or acitrom, are the most widely prescribed oral anticoagulants agents and have been associated more with the development of hematomas than direct factor X inhibitors, such as rivaroxaban [ 1]. Few reports have linked oral anticoagulation therapy with the development of muscular hematomas; however, clinical cases regarding the involvement of the sartorius muscle remain limited. Patients with advanced age, under oral anticoagulant therapy with pain and ecchymosis in the thigh region, should undergo radiological evaluation utilizing ultrasonography, computed tomography or magnetic resonance imaging to establish an accurate diagnosis. The following case consists of a patient that while resting presented with a spontaneous rupture and hematoma of the sartorius muscle secondary to rivaroxaban use. During follow-up, the patient recovered completely.

## INTRODUCTION

The rate of major extra cranial bleeding in patients under oral anticoagulant therapy is between 0.4 and 2% per year [2]. Globally, warfarin is the most frequently prescribed oral anticoagulant and is associated with a higher risk of bleeding compared with the direct factor Xa inhibitors [3]. In the majority of cases, spontaneous muscular hematomas are attributed to oral anticoagulation therapy. Therefore, a high degree of suspicion is necessary when patients present with pain, edema and ecchymosis in a muscle region. Diagnostic imaging studies are compulsory to establish an adequate diagnosis and to decide between conservative and surgical treatment [4]. In few cases, thigh muscles have been affected. However, reports regarding sartorius muscle involvement remain limited. The present case consists of a patient that while resting developed a non-traumatic rupture and hematoma of the sartorius muscle associated to rivaroxaban therapy.

## CASE PRESENTATION

**Figure 1:**
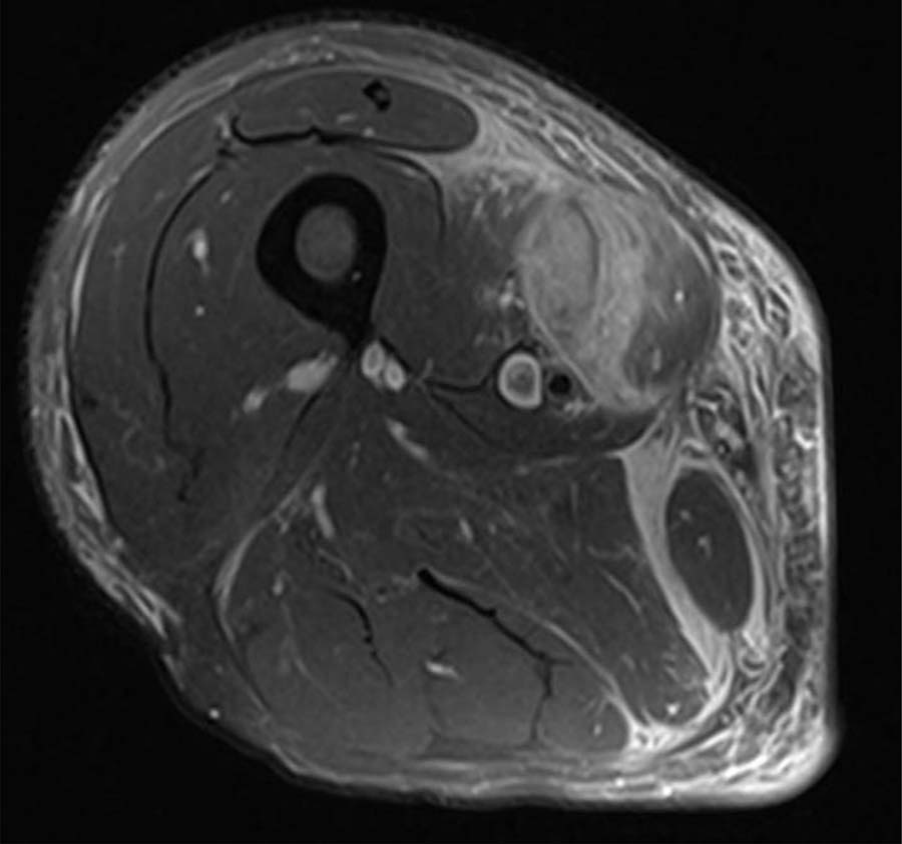
MRI axial view of right thigh region showing hematoma.

**Figure 2:**
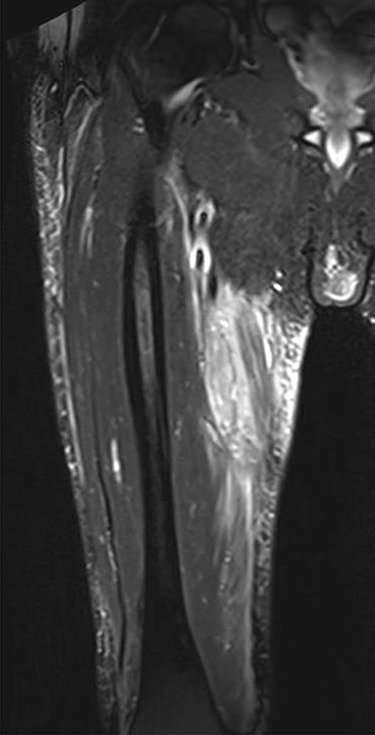
MRI sagittal view manifesting hematoma in right thigh region.

**Figure 3:**
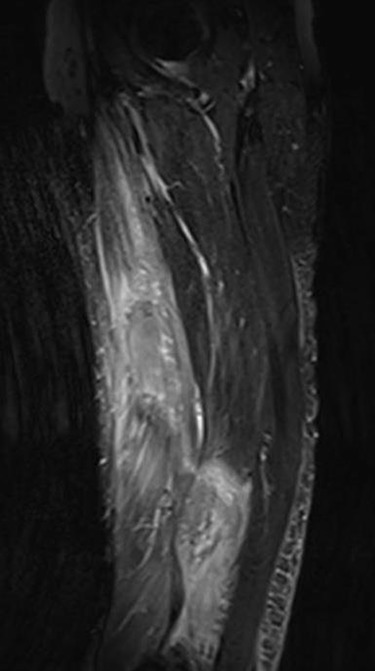
MRI coronal view displaying hematoma and rupture of sartorius muscle.

The clinical case consisted of a 79-year-old male patient with a medical history of atrial fibrillation and pulmonary embolism diagnosed 2 years ago. Among the prescribed medications, rivaroxaban was given as an anticoagulant therapy. The patient was lying in bed when a sudden onset of a sprain-like pain appeared on his right thigh region. He had difficulty walking and observed the progression of a large ecchymosis along the thigh, extending from the greater trochanter of the femur to above the patella. Later as the clinical manifestations worsened, the patient arrived at the emergency department. At the hospital, the treating physician noticed edema, ecchymosis and pain confined to the thigh region; therefore, ordered laboratory studies, such as prothrombin time, international normalized ratio and hemoglobin were all within the normal range for the patient’s age and comorbidities. The doctor solicited a magnetic resonance imaging (MRI) study to evaluate the thigh region where a rupture and hematoma regarding the sartorius muscle was evident. The MRI displayed a hyperintense area representing blood collection and discontinuity in the trajectory of the sartorius muscle (Figs 1–3). The hematoma was large in volume, hence surgical drainage was performed. After the procedure, the patient’s pain diminished considerably and was ordered to rest. Posteriorly, the patient began rehabilitation exercises and cooperated with follow-up dates to test progression. The patient attended a follow-up appointment after 6 months of initial presentation where a positive clinical outcome and enhanced walking capacity was observed.

## DISCUSSION

Since its discovery in 1941, warfarin has been the most commonly prescribed oral anticoagulant agent. Although this coumarin is associated with a reduction of stroke risk, many setbacks have been identified such as unpredictable pharmacokinetics and pharmacodynamics, a slow onset and offset of action, a narrow therapeutic window and variable dose response among each patient [5]. Additionally, the risk of major bleeding is greater with warfarin than non-vitamin K oral anticoagulants (NOACs). Rivaroxaban is a direct factor Xa inhibitor that has the similar efficacy in stroke prevention but a lower incidence in intracranial bleeding than coumarins. However, incidence of gastrointestinal bleeding is slightly higher with rivaroxaban compared to warfarin. Evidently, these drugs provoke an imbalance in the physiology of coagulation factors; therefore, laboratory monitoring of bleeding is necessary [3, 5]. However, in rare instances, bleeding can occur when the patient’s international normalized ratio (INR) and prothrombin time values lie within the therapeutic range such as the patient in the present case.

Rivaroxaban is considered safe when INR values lie between 1.2 and 2.5, nonetheless with a 7% major bleeding risk when the value exceeds 2.5. Intracranial bleeding can occur in certain patients especially when INR values lie above 3; however, limited cases have been reported when plasma levels, INR and prothrombin time are normal [6]. Ideally, NOAC plasma concentration levels can be identified utilizing high-performance liquid chromatography-tandem mass spectroscopy, but such testing is limited to reference laboratories and is unfeasible for routine clinical use. When NOAC concentration levels exceed ideal values, there is an increased risk of bleeding particularly from either an intracranial or gastrointestinal source [6, 7]. Nonetheless, in some instances, muscular hematomas have been reported in patients under anticoagulant treatment. *Çinar et al.* described four cases of spontaneous muscular hematomas. However, they were attributed to warfarin and affected the psoas, quadriceps, pectoral and rectus abdominis muscles [8]. To the best of our knowledge, no report has described a non-traumatic rupture and hematoma localized in the sartorius muscle such as this case.

Thigh hematomas initially present with localized edema, pain and ecchymosis along the affected region. In these cases, radiological imaging using ultrasonography, computed tomography or magnetic resonance imaging is necessary to establish an accurate diagnosis especially in patients taking oral anticoagulant medications. Radiological modalities vary in affordability and image clarity. Although ultrasonography is the most economical, interpretation is user dependent. Small hematomas are treated in a conservative fashion [4, 9]. Conservative treatment consists of rest, ice application, local compression and limb elevation. Nevertheless, large volume of hemorrhages may require surgical drainage and hemoglobin testing to justify the need for blood transfusion. Following initial management, the patient must undergo rehabilitation to optimize range of motion and progressively diminish pain [9].

## CONCLUSION

Spontaneous muscular hematomas in the thigh region are infrequent and are attributed in the majority of cases to oral anticoagulant therapy. This diagnosis must be considered in patients who manifest pain, ecchymosis and edema in the thigh region. Although rivaroxaban treatment is considered safer, treating physicians ought to consider the possibility. In this particular case, the patient was lying in bed and spontaneously developed a rupture and hematoma of the sartorius muscle secondary to rivaroxaban use, which is rather rare since most cases are chiefly linked to warfarin. Clinicians are advised to acknowledge this unusual bleeding site in patients under anticoagulation treatment regardless of drug safety and less variability of response to prevent negative effects in the patient’s quality of life.

## CONFLICT OF INTEREST STATEMENT

None declared.
